# Transcriptomic identification of high-altitude illness candidate genes and physiological mechanisms: a multidimensional bioinformatics study with 106 drug–food homologous substance target prediction

**DOI:** 10.3389/fphys.2026.1752620

**Published:** 2026-10-14

**Authors:** Defang Liu, Jiao Yan, Ting Luo, Yu Guo, Yubo Li, Pan Long, Ling Yang

**Affiliations:** 1 Department of Traditional Chinese Medicine, The General Hospital of Western Theater Command, Chengdu, Sichuan, China; 2 Department of Geriatrics, The General Hospital of Western Theater Command, Chengdu, Sichuan, China; 3 Department of Ophthalmology, The General Hospital of Western Theater Command, Chengdu, Sichuan, China

**Keywords:** bioinformatics, biomarker, drug-food homologous substance, environmental physiology, high-altitude illness, mechanisms

## Abstract

**Introduction:**

High-altitude (HA) illness threatens health after acute high-altitude exposure, but its molecular mechanisms and effective interventions remain unclear. Drug–food homologous (DFH) substances have potential for HA illness intervention, yet their active components and targets are unknown.

**Methods:**

Datasets related to HA illness, as well as 106 DFH substances and their corresponding chemical components, were obtained in this study. Differentially expressed genes were identified, and active components were screened according to the criteria of drug-likeness ≥0.18 and oral bioavailability ≥30%. The GraphBAN tool was used to predict high-confidence compound–protein interaction pairs, so as to obtain the corresponding candidate active components and candidate genes. Meanwhile, the ADMETlab3 platform was employed to evaluate the druggability and toxicity of these active components, thereby determining the core active components and core genes. Candidate genes were screened were screened out by machine learning, and relevant interaction networks were constructed. Finally, further studies such as pathway enrichment, immune infiltration, molecular docking, and molecular dynamics simulation were carried out.

**Results:**

Of the 95 candidate active components subjected to toxicological screening, only 8 passed the safety evaluation, and 6 of these met both physicochemical and toxicological criteria; five candidate genes (DNAJC19, MRFAP1L1, TBPL1, RPS21, and NABP1) and two key active components (polyacetylene PQ-2, PQ-2) were identified, and these candidate genes corresponded to the DFH substance “*Panacis Quinquefolii Radix*”. The abovementioned candidate genes were enriched in pathways such as ribosome and oxidative phosphorylation, and they were correlated with most differential immune cells. Except for RPS21 and MRFAP1L1, the binding abilities of the other candidate genes with the key active components were good (binding energies <−5.0 kcal/mol). Molecular dynamics simulation confirmed that the interaction of their binding was stable.

**Discussion:**

*Panacis Quinquefolii Radix* might intervene in HA illness via active components (PQ-2) that target five candidate genes, regulating immunity and metabolism. This provided a preliminary computational basis for DFH-based HA illness prevention/treatment.

## Graphical Abstract

**Figure:**
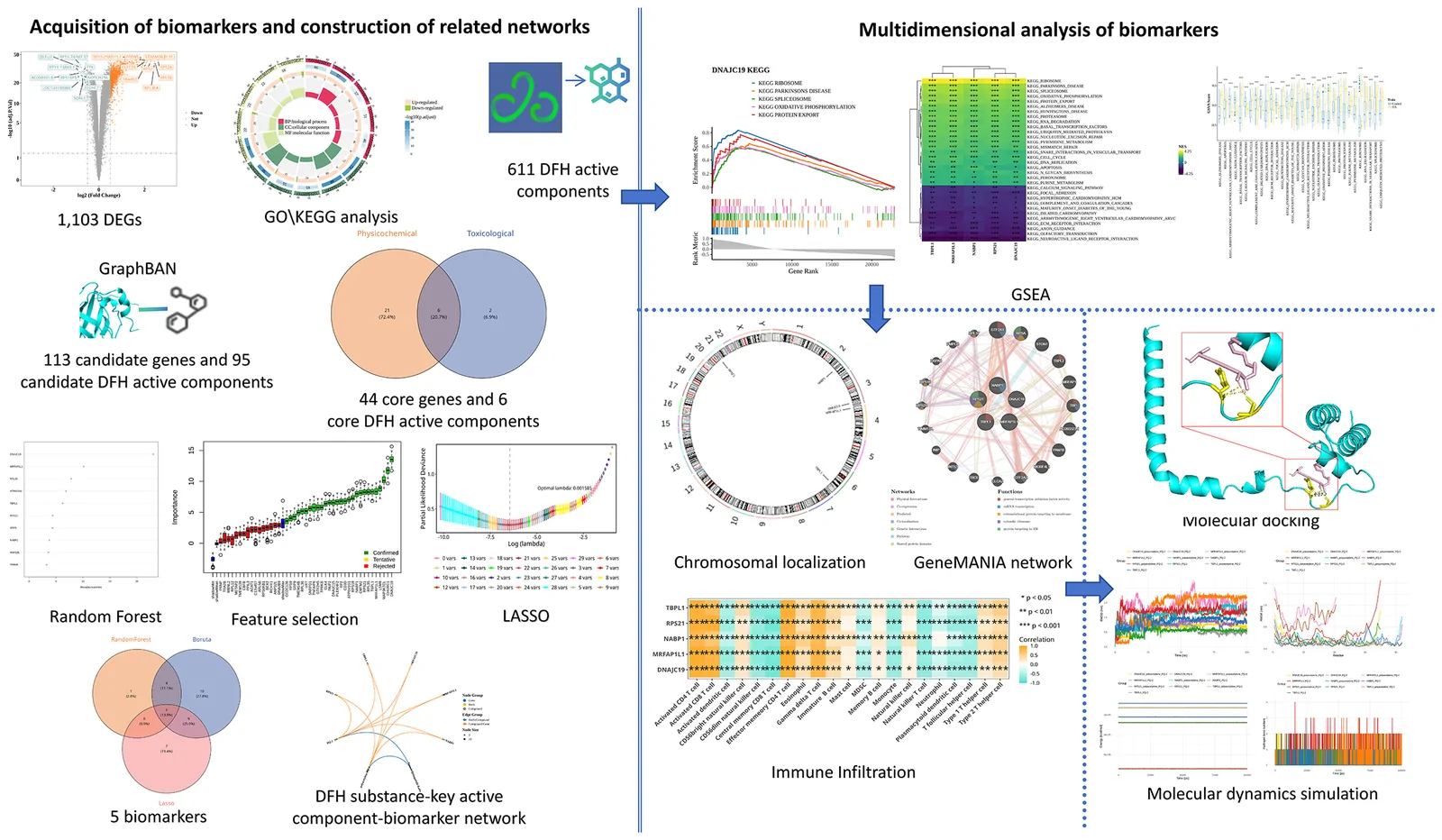

## 1 Introduction

High-altitude (HA) conditions are characterized by high elevation, low atmospheric pressure, intense ultraviolet radiation, and hypoxia (Betge et al., 2020). Rapid ascent to HA triggers stress responses, often causing HA illness in unacclimated individuals from lowland areas (Richalet et al., 2024). Common HA illnesses include acute mountain sickness (AMS), high-altitude cerebral edema (HACE), and high-altitude pulmonary edema (HAPE) (Li et al., 2024; Chaoyi et al., 2026). Prolonged HA exposure can reversibly or irreversibly damage oxygen-sensitive tissues, including the brain, heart, liver, gastrointestinal tract, and visual system (Dhar et al., 2014; Gustavo F and Vilma, 2014; Luks and Swenson, 2015; Willmann et al., 2014; Zhang et al., 2018), and may even be lethal (Tianyi et al., 2006). The pathogenesis involves hypoxic stress, inflammation, mitochondrial dysfunction, and immune imbalance (Li et al., 2023; Mallet et al., 2023; Zhou et al., 2025). Recent studies have further revealed that AMS-susceptible individuals cannot effectively inhibit the tricarboxylic acid cycle, glycolysis, or protein synthesis and degradation under hypoxic conditions, thereby failing to reduce oxygen consumption and energy expenditure (Hui et al., 2018). Moreover, mitochondrial haplogroup M33a2’3 and its associated conserved variants (e.g., G4491A and A4944G of MT-ND2, and A14002G of MT-ND5) may reduce protein stability, constituting a molecular basis for susceptibility to HAPE (Swati et al., 2019). However, the core molecular pathways and key regulatory genes remain poorly defined, limiting the development of precise diagnostic biomarkers and targeted therapies. Systematically identifying differentially expressed genes (DEGs) from biological samples (e.g., peripheral blood mononuclear cells, PBMCs) exposed to HA conditions can therefore help elucidate the underlying pathological mechanisms.

The concept of drug and food homology (DFH) originates from the medical tradition of *Huang Di Nei Jing*, which advocates “using medicinals to dispel pathogens, followed by food therapy to support recovery”. To standardize and promote the scientific application, the Chinese National Health Commission has officially recognized 106 natural substances that serve both culinary and medicinal purposes. Traditional Chinese medicine principles such as replenishing qi and nourishing yin, promoting blood circulation and resolving stasis, and regulating metabolism (Yaqun et al., 2025) align closely with the pathological features of HA conditions characterized by hypoxic injury, metabolic dysregulation, and immune imbalance. Active components of certain DFH substances (e.g., ginkgo, licorice, and angelica) have demonstrated antihypoxic, anti-inflammatory, and cardiopulmonary-protective effects (Caini et al., 2025; Lulu et al., 2025; Xiaodong et al., 2024). However, most studies remain limited to *in vitro* or animal models focusing on single ingredients, and a systematic analysis of the “component–target–disease” network is lacking. Moreover, research on DFH substances for HA illnesses has yet to integrate human transcriptomic data for precise target identification or to comprehensively evaluate the druggability and toxicological profiles of these components, impeding clinical translation.

Hence, this study aimed to integrate network pharmacology and transcriptomics to screen for HA-related DEGs across the transcriptome, identifying key biological processes, signaling pathways, and candidate genes impacted by bioactive DFH components. This systematic approach addresses the mechanistic gap in DFH-based interventions for HA illnesses. Importantly, PBMC transcriptomics during acute high-altitude exposure directly reveals hypoxia-induced immune dysregulation and metabolic reprogramming, central to environmental physiology. We expect that our findings will identify diagnostic candidate genes for early HA illness detection and propose candidate compounds for adjunct therapy, thereby advancing the modern application of traditional Chinese medicine in HA illness management.

## 2 Materials and methods

### 2.1 Dataset retrieval and drug–food homologous substance compilation

The HA-related dataset GSE46480 was retrieved from the Gene Expression Omnibus (GEO) database (https://www.ncbi.nlm.nih.gov/geo/). It comprised 98 PBMC samples from healthy volunteers after acute exposure to altitude (HA group), and 98 PBMC samples from healthy volunteers at sea level (control group) were included in the dataset, which were mainly used for differential expression analysis and other related studies. The sequencing platform was GPL570, which corresponds to the Affymetrix Human Genome U133 Plus 2.0 Array—a high-throughput microarray platform for human whole-genome transcript expression profiling that covers more than 38,500 annotated human genes and 47,000 transcripts. Meanwhile, the 106 DFH substances were obtained in accordance with the Official List of Food and Medicinal Homologous Substances released by the National Health Commission (NHC) of the People’s Republic of China. The English official website of NHC is available at https://en.nhc.gov.cn/; the full list can be retrieved by searching the English keyword List of Food and Medicinal Homologous Substances on the English portal (**Supplementary Table 1**).

### 2.2 Identification and analysis of differentially expressed genes

Raw microarray expression data were downloaded from the GEO database using the GEOquery R package. Standard preprocessing of the Affymetrix GPL570 microarray data was conducted before differential analysis: the expression matrix was log2(expr + 1) transformed if not in the logarithmic scale; probe sets were annotated using the platform annotation file, with unannotated probes discarded; for multiple probes mapping to the same gene symbol, the probe with the highest mean expression was selected as the gene representative. The “limma” package (v 3.56.2) (Shiyi et al., 2021) was used to screen DEGs between the HA group and the control group in the dataset, with the criteria of |log_2_FoldChange (FC)| > 0.5 and adjusted *p*.value < 0.05. A volcano plot of DEGs was generated using the “ggplot2” package (v 3.5.2) (Jinxiang et al., 2023), and the top 10 genes with the largest upregulation and downregulation were labeled. Additionally, a heatmap of these labeled genes was created using the “ComplexHeatmap” package (v 2.16.0) (Zuguang, 2022).

Gene Ontology (GO) and Kyoto Encyclopedia of Genes and Genomes (KEGG) enrichment analyses were performed with the “clusterProfiler” package (v 4.8.1) (Zizheng et al., 2022) at adjusted *p* < 0.05. The top five results were presented based on the ranking of gene enrichment ratios.

Furthermore, amino acid sequences (protein sequences) of DEGs were retrieved from the UniProt database (https://www.uniprot.org) via the “biomaRt” package (v 2.56.1) (Steffen et al., 2009).

### 2.3 Network pharmacology

Chemical components of the 106 DFH substances were retrieved from the Traditional Chinese Medicine Systems Pharmacology Database and Analysis Platform (TCMSP, English official site: https://www.tcmsp-e.com/tcmsp.php). The English names of each DFH substance were input into the Herb search box on the English interface for component retrieval. Active components were screened using the criteria of drug-likeness (DL) ≥ 0.18 and oral bioavailability (OB) ≥ 30%, and their Mol IDs and molecular structure were recorded. Subsequently, the Simplified Molecular-Input Line-Entry System (SMILES) structures of the corresponding DFH active components were obtained from the PubChem database (https://pubchem.ncbi.nlm.nih.gov/) by combining the PubChem CIDs provided by TCMSP.

To screen candidate DFH active components and candidate genes with potential intervention effects on HA, GraphBAN, a bioinformatics analysis tool developed based on graph neural networks for efficient and accurate prediction of compound–protein interactions (CPI), was adopted. Based on three pretrained models from BindingDB, BioSNAP, and KIBA, the interaction probabilities between the amino acid sequences of DEGs and DFH active components were predicted (P(pred) > 0.7). This cutoff (P(pred) > 0.7) is the official recommended high-confidence screening threshold of the GraphBAN tool, and it is also a universal threshold adopted in similar CPI prediction studies to ensure high reliability of the screened CPI pairs and avoid false-positive results. The prediction results of the three pretrained models were intersected to obtain highly reliable CPI pairs, and thus candidate DFH active components and candidate genes were identified.

Druggability of the candidate DFH active components was evaluated using the ADMETlab3 platform (https://admetlab3.scbdd.com/server/screening). First, this platform was employed to calculate the core physicochemical parameters of candidate DFH active components, including molecular weight (MW), number of hydrogen bond acceptors (HBA), number of hydrogen bond donors (HBD), and Logarithm of the Partition Coefficient (LogP). These parameters were used to evaluate the compliance of candidate DFH active components with the Lipinski’s Rule of Five. Generally, the criteria for compliance were set as MW < 500, HBA < 10, HBD ≤ 5, and LogP ≤ 5. Fewer violations indicate higher oral bioavailability and better druggability. The topological polar surface area (TPSA) was also calculated; values < 140 Å² suggest good cell membrane permeability, favoring oral absorption. A multistage progressive screening strategy was employed. First, 96 candidate compounds were screened for physicochemical properties. Second, toxicity properties including hERG, H-HT, DILI, carcinogenicity, and ROA were evaluated. Compounds with probability < 0.5 for all toxicity endpoints were retained.

In addition, toxicological properties were also assessed on ADMETlab3, including human hepatotoxicity (H-HT), human ether-a-go-go-related gene (hERG) potassium channel inhibitory toxicity, drug-induced liver injury (DILI), carcinogenicity, and acute oral toxicity in rats. Compounds were retained only if the predicted probability for each toxicity endpoint was below 0.5. For compounds sharing identical molecular formulas but differing in stereochemical configuration, comparative structural alignment and ADMET profiling were performed to evaluate the potential influence of stereoconfiguration on physicochemical properties and toxicity endpoints. Those that satisfied both the physicochemical and toxicological criteria were designated as core DFH active components, and their corresponding candidate genes as core genes. To prioritize candidates for mechanism-centered analyses, these core active components were further intersected with high-confidence compound–protein interaction pairs involving the final biomarkers identified through machine learning-based feature selection. Only core components exhibiting predicted interactions with these biomarkers were retained as key active components for downstream network construction, docking, and molecular dynamics analyses; core components lacking biomarker-directed predictions were excluded from subsequent target-specific mechanistic investigations.

### 2.4 Identification of candidate genes and construction of the DFH substance-key active component-candidate gene network

To identify biomarkers, core genes underwent a three-step feature selection within the dataset. First, a random forest (RF) model (number of trees, 20) was constructed using the “randomForest” package (v 4.7.1.2) (Yao et al., 2023), and genes were ranked by importance to obtain the first feature set. Second, the “Boruta” package (v 8.0.0) (Neha Shree et al., 2023) selected a second set of features with a “confirmed” status. Third, least absolute shrinkage and selection operator (LASSO) regression was performed with the “glmnet” package (v 4.1.8) (Ying et al., 2022) using 10-fold cross-validation; feature genes retained at the optimal lambda (lambda.min) constituted the third set. The intersection of these three feature sets, obtained via the “ggvenn” package (v 0.1.10) (Wenchao et al., 2025), defined the final, candidate genes closely related to HA and 106 DFH substances.

To evaluate the functional specificity and biological relevance of the feature genes selected by each algorithm, we performed separate GO enrichment analyses on the gene sets derived from RF, Boruta, and LASSO. The “clusterProfiler” package (v 4.8.1) was used with p value < 0.05 as the significance threshold.

Key active components corresponding to these candidate genes were then identified from the high-confidence CPI pairs. The DFH substances corresponding to the key active components were determined, and the DFH substance-key active component-candidate gene network was further constructed using the “ggraph” package (v 2.2.1) (Noriaki et al., 2023).

### 2.5 Construction and evaluation of a predictive model based on biomarkers

To explore the predictive capability of biomarkers for the probability of HA occurrence, we constructed a nomogram model using the GSE46480 dataset. The nomogram was developed using the “rms” package (v 6.5-0) (https://cran.r-project.org/package=rms). Calibration curves were generated using the “rms” package (v 6.5-0) to assess the agreement between predicted probabilities and observed outcomes, with 1,000 bootstrap resampling iterations for internal validation. The receiver operating characteristic (ROC) curve was plotted using the “pROC” package (v 1.18.5) (Robin et al., 2011), and the area under the curve (AUC) with 95% confidence interval (CI) was calculated to quantify the model’s discriminative performance. Additionally, decision curve analysis (DCA) was performed using the “ggDCA” package (v 1.2) (https://github.com/yikeshu0611/ggDCA) to evaluate the clinical net benefit of the nomogram across a range of threshold probabilities.

### 2.6 Multidimensional analysis of candidate genes

To explore the biological pathways of candidate genes in HA, Spearman correlation coefficients between candidate genes and all genes in the dataset were calculated using the “psych” package (v 2.5.3) (Rizqy Amelia and Hanif, 2024), and genes were ranked in descending order. Gene set enrichment analysis (GSEA) was then performed with the “clusterProfiler” package (v 4.8.1) against c2.cp.kegg_legacy.v2025.1.Hs.symbols.gmt gene set from the Molecular Signatures Database (MSigDB, http://www.gsea-msigdb.org/gsea/msigdb/). Pathways with |Normalized Enrichment Score (NES)| > 1, *p* < 0.05, and *p*.adjust < 0.05 were considered significant, and the top 5 were ranked by NES. The intersection of pathways enriched by individual candidate genes identified the core pathways commonly involved. A cluster heatmap visualized the enrichment scores of these shared pathways, and differences between the HA group and control group were assessed using the “GSVA” package (v 1.48.2) (Yi et al., 2023) with the Wilcoxon rank-sum test (*p* < 0.05).

To determine the specific positions of candidate genes on chromosomes, the “RCircos” package (v 1.2.2) (Hongen et al., 2013) was used for chromosome localization of candidate genes. Moreover, to explore the interactions and functional associations between candidate genes and other human genes, the GeneMANIA database (https://genemania.org) was used to predict the interaction relationships between candidate genes and potentially related genes.

Studies have shown that immune infiltration is closely related to the occurrence and development of HA (Qiong et al., 2023). Therefore, to study the immune cell infiltration during HA, the HA group and the control group were analyzed based on the dataset. The input data for ssGSEA were not absolute gene expression counts, but the standardized gene expression matrix of the Affymetrix microarray after RMA normalization (rows represent genes, columns represent samples), which had undergone background correction, quantile normalization, and log2 transformation, consistent with the data used for previous differential expression analysis. Specifically, the signature gene sets for 28 immune cell types were obtained from the supplementary mmc1 file of a published study (Gabriela et al., 2013). The ssGSEA algorithm in the “GSVA” package (v 1.48.2) was used to evaluate the relative abundance of 28 types of immune cells, with core parameters set as follows: method, “ssgsea,” kcdf, “Gaussian” (suitable for normalized continuous expression data), normalization within samples was performed, and the enrichment score of each immune cell was calculated independently for each sample, finally outputting an enrichment score matrix of the sample × immune cell. The Wilcoxon rank-sum test was used to compare the differences in immune cells between the two groups, and immune cells with *p* < 0.05 were defined as differential immune cells (DICs). The “psych” package (v 2.5.3) was used to explore the correlations between DICs and between candidate genes and DICs. A significant correlation was defined as correlation (cor)| > 0.3, and adjusted *p* < 0.05 (Benjamini–Hochberg FDR correction). *P* values were adjusted using the Benjamini–Hochberg (BH) method to control the false discovery rate (FDR).

Finally, to clarify the interaction between key active components and their corresponding candidate gene proteins, molecular docking was performed; protein structures (Protein Data Bank (PDB) files) were obtained from the AlphaFold Protein Structure Database (https://alphafold.com/), and the three-dimensional structures (Structural Data File (SDF) files) of key active components were obtained from the PubChem database. These two types of structures were imported into the online tool CB-DOCK2 (https://cadd.labshare.cn/cb-dock2/php/index.php) for docking. Generally, a binding energy < −5.0 kcal/mol is considered to indicate good binding activity.

To validate the docking results, 100 ns molecular dynamics simulation were performed using GROMACS software (v S2024.2) (Dongping et al., 2024). Target protein sequences (human species) were retrieved from the National Center for Biotechnology Information (NCBI, https://www.ncbi.nlm.nih.gov/) database. The AMBER14SB force field was applied to proteins, and the AMBER GAFF force field to small-molecule ligands. Periodic boundary conditions were set, the simulation box was optimized, and the system was solvated with water molecules. Na^+^ and Cl^-^ ions (0.15 mol/L) were added to neutralize the system. Following energy minimization via the steepest descent method, two-stage pre-equilibration was performed: an NVT system (constant number of particles, volume, and temperature) for simulation at 300 K for 100 ps to stabilize the system temperature; followed by an NPT system (constant number of particles, pressure, and temperature) for simulation at 1 bar for 100 ps to stabilize the system pressure, laying a stable foundation for subsequent simulations. After equilibration, the system entered the production simulation stage. A total of 10 candidate gene–ligand complexes were subjected to this simulation, specifically DNAJC19_polyacetylene_PQ−2; DNAJC19_PQ−2; MRFAP1L1_polyacetylene_PQ−2; MRFAP1L1_PQ−2; NABP1_polyacetylene_PQ−2; NABP1_PQ−2; RPS21_polyacetylene_PQ−2; RPS21_PQ−2; TBPL1_polyacetylene_PQ−2; and TBPL1_PQ−2. To comprehensively evaluate the structural changes and interaction characteristics of the protein–ligand complexes, key parameters were calculated: root mean square deviation (RMSD, nm), root mean square fluctuation (RMSF, nm) of protein residues, total system energy (kJ/mol), number of hydrogen bonds, and MM-PBSA binding free energy.

### 2.7 Statistical analysis and tool versions

Using the R programming language (v 4.2.2), all analyses were conducted. *p* < 0.05 was considered statistically significant. Cohort comparisons were made using the Wilcoxon rank-sum test.

All analyses were performed using the following software, tools, and R packages with exact versions: R programming language: v4.2.2; limma: v3.56.2; ggplot2: v3.5.2; ComplexHeatmap: v2.16.0; clusterProfiler: v4.8.1; biomaRt: v2.56.1; randomForest: v4.7.1.2; Boruta: v8.0.0; glmnet: v4.1.8; ggvenn: v0.1.10; ggraph: v2.2.1; psych: v2.5.3; GSVA: v1.48.2; RCircos: v1.2.2; GROMACS: vS2024.2; GraphBAN: graph neural network-based CPI prediction tool; ADMETlab3: online ADMET evaluation platform; CB-DOCK2: online molecular docking tool; Sequencing platform: Affymetrix Human Genome U133 Plus 2.0 Array (GPL570).

## 3 Results

### 3.1 Acquisition and enrichment analysis of DEGs during HA progression

A total of 1,103 DEGs were screened from the dataset with the criteria of |log_2_FC| > 0.5 and adjusted *p*.value < 0.05. Among these, compared with the control group, 1,063 genes were upregulated and 40 genes were downregulated in the HA group (**Supplementary Table 2**). The volcano plot of DEGs and the heatmap of labeled genes are shown in **Figures 1A, B** respectively. Enrichment analysis was performed based on these DEGs with the criterion of *p*.adjust < 0.05. The results indicated that a total of 220 functional terms were obtained from GO enrichment analysis, including 108 terms in the biological process (BP), 92 terms in the cellular component (CC), and 20 terms in molecular function (MF). These terms involved multiple important functions, such as ribonucleoprotein complex biogenesis (GO:0022613), cytoplasmic translation (GO:0002181), ribosome (GO:0005840), ribosomal subunit (GO:0044391), structural constituent of ribosome (GO:0003735), and electron transfer activity (GO:0009055) (**Supplementary Table 3**; **Figure 1C**). KEGG enrichment analysis covered 20 pathways, including pathways related to neurodegeneration-multiple diseases (hsa05022) (**Supplementary Table 4**; **Figure 1D**). The “cytoplasmic translation” (GO:0002181) enriched by DEGs was directly associated with protein synthesis disorders induced by HA hypoxia, whereas the “neurodegeneration-multiple diseases” pathway (hsa05022) might be involved in central nervous system damage caused by HA. Both provided a pathological direction for the subsequent screening of HA-related targets.

**Figure 1:**
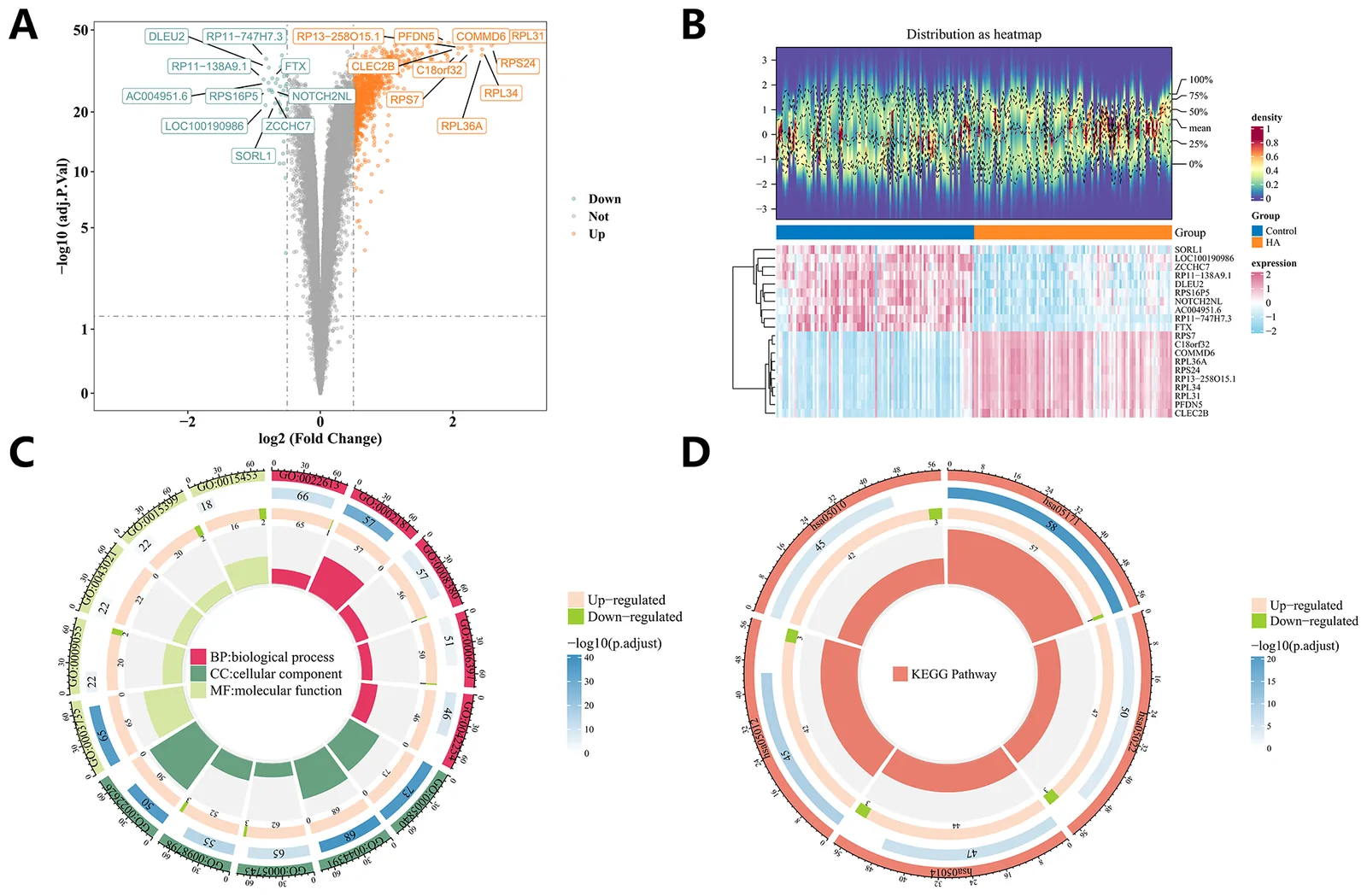
Identification of DEGs and enrichment analysis. **(A)** Volcano plot of DEGs between the high-altitude (HA) group and the control group. DEGs were screened with |log2FC| > 0.5 and adjusted *p*.value < 0.05; orange: upregulated DEGs, green: downregulated DEGs, gray: no significant difference genes. **(B)** Heatmap of top 10 upregulated and downregulated DEGs. Central bar: blue, control group, orange, HA group; color scale: pink, high gene expression, blue, low gene expression; top density plot indicates gene expression abundance. **(C)** GO enrichment bubble chart of DEGs. Outermost ring: GO term ID; second ring: number of enriched genes (color depth, significance); third ring: count of upregulated (orange) and downregulated (green) genes; innermost ring: GO category (biological process, cellular component, molecular function). **(D)** KEGG enrichment bubble chart of DEGs. Outermost ring: KEGG pathway ID; second ring: number of enriched genes (color depth, significance); third ring: count of upregulated (orange) and downregulated (green) genes; innermost ring: KEGG pathway classification. DEGs, differentially expressed genes; GO, Gene Ontology; KEGG, Kyoto Encyclopedia of Genes and Genomes; FC, fold change.

### 3.2 Screening of core DFH active components and core genes

Based on 106 DFH substances, 610 DFH active components with good bioavailability and potential activity were screened from TCMSP using the criteria of DL ≥ 0.18 and OB ≥ 30% (**Supplementary Table 5**). Subsequently, based on the previously obtained SMILES of DFH active components and amino acid sequences of DEGs, three sets of high-confidence CPI pairs were screened using three pretrained models (BindingDB, BioSNAP, and KIBA) with the criterion of P(pred) > 0.7 (**Supplementary Tables 6**–**8**). By taking the intersection of the prediction results from the three models, 754 highly reliable CPI pairs were obtained, which included 113 candidate genes and 95 candidate DFH active components (**Supplementary Table 9**).

Next, the physicochemical properties of the 95 screened candidate DFH active components were evaluated. It was found that among these 95 compounds, 27 candidate DFH active components met the set screening thresholds for core physicochemical parameters, with no violations of Lipinski’s Rule of Five, and all exhibited good cell membrane permeability, showing high overall druggability potential. **Table 1** presents the corresponding physicochemical property evaluation results. Meanwhile, the toxicological properties of the 95 candidate DFH active components were assessed, and the results showed that 8 candidate DFH active components passed the toxicological safety evaluation (**Table 2**) representing a high elimination rate in toxicological screening with no significant toxicity risks. Moreover, some components showed outstanding performance in dimensions such as cardiac safety, liver protection, and low carcinogenicity, providing a reliable safety basis for the subsequent screening of core components that meet both “physicochemical property + toxicology” standards and the verification of HA intervention potential. On this basis, there were six candidate DFH active components that met both the physicochemical property evaluation and toxicological property evaluation criteria, with their corresponding MOL_IDs being MOL011435, MOL011434, MOL000273, MOL000290, MOL000291, and MOL000280 (**Supplementary Figure 1**). Meanwhile, there were 44 candidate genes corresponding to these active components (**Supplementary Table 10**). Finally, the six core DFH active components and the 44 core genes were identified. When these core components were further screened against the biomarker-specific high-confidence CPI pairs, only two retained predicted interactions with the biomarkers and were designated as key active components for subsequent mechanistic analyses; the remaining four, despite favorable ADMET profiles, lacked biomarker-directed predictions and were consequently excluded from downstream target-specific investigations. Among the two key active components, PQ-2 and polyacetylene PQ-2 demonstrated favorable ADMET profiles. Both compounds satisfied Lipinski’s Rule of Five (MW < 500, logP < 5, H-bond acceptors < 10, H-bond donors ≦ 5). Notably, despite their similar overall profiles, a modest difference was observed in lipophilicity (LogP: 2.943 for PQ-2 vs. 2.821 for polyacetylene PQ-2), along with slight variations in specific toxicity endpoints (e.g., hERG: 0.081 for PQ-2 vs. 0.059 for polyacetylene PQ-2; hepatotoxicity: 0.313 for PQ-2 vs. 0.289 for polyacetylene PQ-2), suggesting that their distinct stereoconfigurations may subtly influence their physicochemical and binding properties. To clarify the chemical identity of these components, we identified that “PQ-2” (MOL011435) and “polyacetylene PQ-2” (MOL011434) are known polyacetylene compounds derived from *Panacis Quinquefolii Radix*, and they are a pair of epimers (diastereoisomers) with distinct stereochemical configurations. Existing studies on the antihypoxic effects of *Panacis Quinquefolii Radix* are predominantly focused on ginsenosides, and no reports have described the antihypoxic activity of PQ-2 or polyacetylene PQ-2 prior to this work. The detailed chemical information is listed in **Supplementary Table 11**.

**Table 1:** Results of physicochemical property evaluation of 27 candidate DFH active components.

MOL_ID	MW	HBA	HBD	TPSA	logP
MOL000290	498.33	5	3	94.83	4.08186660294751
MOL001521	486.33	5	3	94.83	3.44923198265506
MOL008034	486.33	5	3	94.83	3.40874164952647
MOL000285	482.34	4	2	74.6	3.82994209177001
MOL000280	484.36	4	3	77.76	4.31697392097862
MOL003901	434.34	4	2	58.92	4.40883280905747
MOL008397	436.26	4	0	52.6	3.40606986118255
MOL003889	446.34	4	2	58.92	3.65017816448422
MOL011435	306.18	4	3	73.22	2.94295897067871
MOL011434	306.18	4	3	73.22	2.82125503830758
MOL000291	484.32	5	3	94.83	4.39235413935342
MOL009760	432.32	4	2	58.92	3.34988907034303
MOL007640	416.33	3	2	49.69	4.9505724094385
MOL000273	470.34	4	3	77.76	4.51313869146186
MOL003891	414.31	3	2	49.69	4.84239640724093
MOL005360	432.21	7	2	102.29	2.29191469246731
MOL005458	444.32	4	2	58.92	3.85365851728116
MOL006472	330.07	7	3	113.29	3.10078478626783
MOL012986	472.36	4	2	58.92	4.41469428179627
MOL005088	312.12	7	4	119.75	-0.472902151
MOL009615	456.4	2	2	40.46	4.18247660074344
MOL010387	430.31	4	2	58.92	4.00126326407908
MOL006327	442.38	2	2	40.46	4.93116628257179
MOL011363	300.08	8	5	136.68	0.0590258446078884
MOL011455	418.24	6	3	104.06	2.82333170528185
MOL003014	384.25	6	1	74.22	3.9780368613616
MOL009465	430.31	4	2	74.6	3.44481183058488

**Table 2:** Results of toxicological property evaluation of 8 candidate DFH active components.

MOL_ID	hERG	H-HT	DILI	Carcinogenicity	ROA
MOL000290	0.010	0.473	0.155	0.394	0.161
MOL000280	0.017	0.442	0.045	0.473	0.227
MOL000276	0.015	0.466	0.234	0.484	0.223
MOL010072	0.005	0.294	0.395	0.071	0.028
MOL011435	0.081	0.313	0.409	0.317	0.453
MOL011434	0.059	0.289	0.347	0.392	0.435
MOL000291	0.013	0.439	0.130	0.294	0.185
MOL000273	0.026	0.442	0.063	0.486	0.220

### 3.3 Acquisition of candidate genes related to 106 DFH substances in HA and construction of the DFH substance-key active component-candidate gene network

Based on the core genes, feature selection and LASSO regression analysis were conducted, and three sets of feature genes were successfully screened. As shown in **Figure 2A**, when the number of trees was 20, the error of the RF model was minimized, and the top 10 genes ranked by importance (DNAJC19, MRFAP1L1, RPL35, ATP6V1G1, TBPL1, RPS21, SRP9, NABP1, ANP32E, and TPRKB) were selected as the first set of feature genes. As shown in **Figure 2B**, there were 28 feature genes with the status of “confirmed” (the second set of feature genes; **Supplementary Table 12**). As shown in **Figure 2C**, the model reached the optimal state when the optimal lambda was 0.001585. In this optimal model, a total of 21 feature genes were included (the third set of feature genes; **Supplementary Table 13**). By taking the intersection of the above three sets of feature genes, five candidate genes were finally obtained (**Figure 2D**), namely, DNAJC19, MRFAP1L1, TBPL1, RPS21, and NABP1. These five candidate genes corresponded to two key active components (polyacetylene PQ-2, PQ-2) and one DFH substance (*Panacis Quinquefolii Radix*). Furthermore, the constructed DFH substance-key active component-candidate gene network is shown in **Figure 2E**.

**Figure 2:**
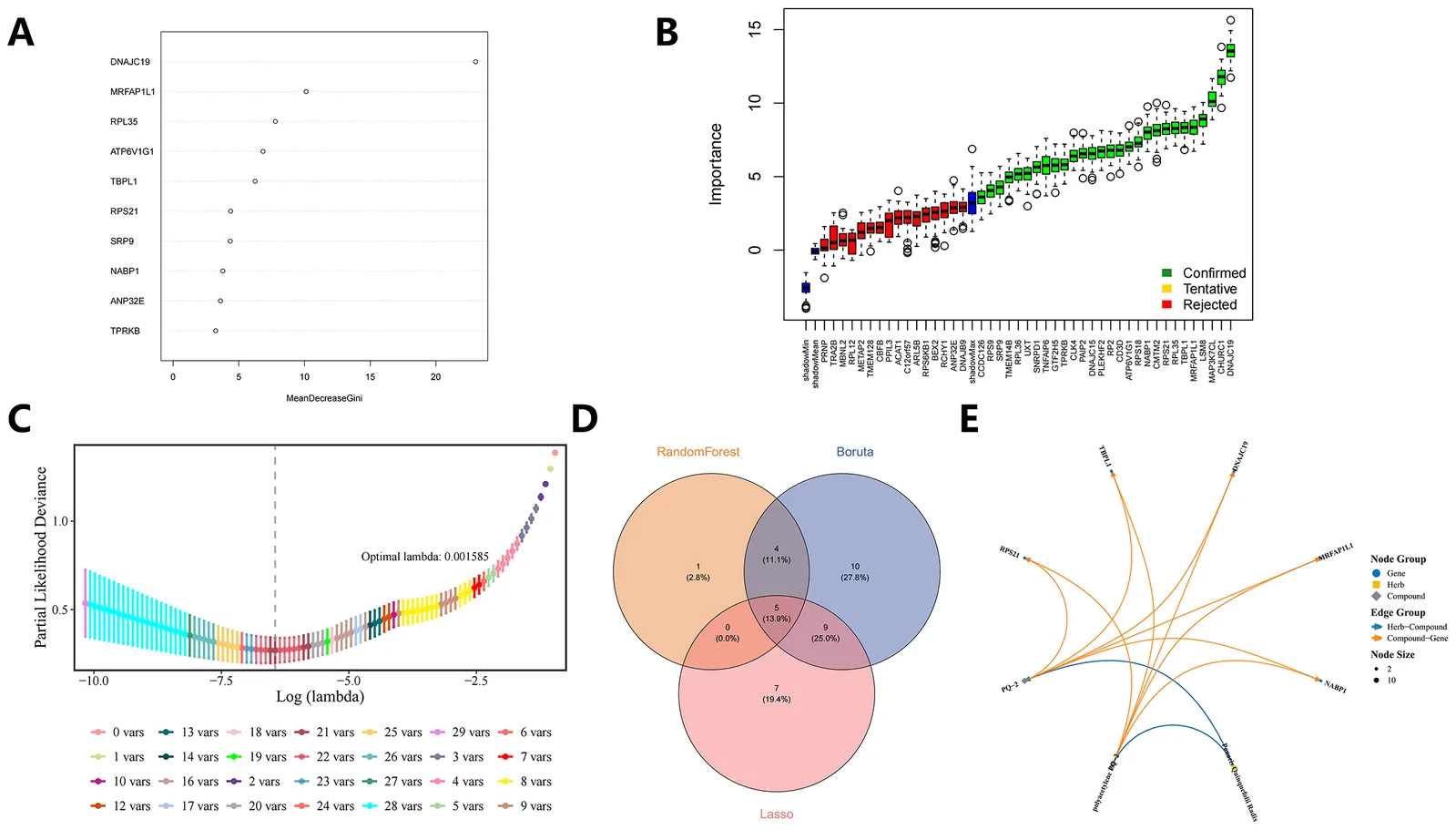
Identification of candidate genes and the underlying regulatory network. **(A)** Importance score of feature genes screened by the random forest model. Top 10 feature genes ranked by MeanDecreaseGini value. **(B)** Feature importance evaluation by the Boruta algorithm. X-axis: gene variables and shadow features; Y-axis: importance score; blue, shadow feature statistics, red, unimportant genes, green, confirmed important genes. **(C)** Parameter optimization of LASSO regression via 10-fold cross-validation. X-axis: log(lambda), Y-axis: partial likelihood deviance; vertical dotted line indicates optimal lambda value. **(D)** Venn diagram of feature genes from three machine learning methods. Overlapping region represents the final five candidate genes. **(E)** Drug–food homologous (DFH) substance-key active component-candidate gene network. Node type: circle, DFH substance, square, key active component, triangle, candidate gene; node size, degree of connectivity; edge, molecular interaction; color scale represents interaction strength. LASSO, least absolute shrinkage and selection operator.

To validate that the five intersecting biomarkers (DNAJC19, MRFAP1L1, TBPL1, RPS21, and NABP1) are functionally more relevant to HA than genes uniquely selected by a single algorithm, we performed separate GO enrichment analyses on the feature genes obtained from RF (top 10), Boruta (28 confirmed genes), and LASSO (21 genes). The results showed that all three algorithms significantly enriched several common core pathways, including “cytosolic ribosome” (GO:0022626), “ribosomal subunit” (GO:0044391), “structural constituent of ribosome” (GO:0003735), and “cytoplasmic translation” (GO:0002181) (**Supplementary Figure 2**; **Supplementary Table 14**), all of which are closely associated with hypoxia-induced energy deficits and impaired protein synthesis.

To further validate the robustness of these five identified candidate genes, we utilized two independent external datasets, GSE196728 and GSE133702. In the GSE196728 dataset (comparing samples at 3,800 and 5,100 m), although the five candidate genes showed consistent expression trends with the discovery dataset, they did not reach statistical significance (**Supplementary Figure 3**). Subsequently, we performed validation using the GSE133702 dataset (comparing Basal at 800 m and Altitude at 4,111 m). As shown in **Supplementary Figure 3**, RPS21 showed a significant differential expression (*p* < 0.05). Additionally, DNAJC19 and NABP1 showed marginal significance (0.05 < *p* < 0.1) and a consistent downregulation trend under high-altitude conditions. To further evaluate cross-cohort robustness, a random-effects meta-analysis of the two external datasets was performed using Hedges’ g. The pooled analysis revealed statistically significant downregulation for MRFAP1L1 (SMD, −0.732, 95% CI: −1.223 to −0.242, P, 0.003), TBPL1 (SMD, −0.988, 95% CI: −1.490 to −0.485, P, 1.16 × 10^-^^4^), and NABP1 (SMD, −1.108, 95% CI: −1.616 to −0.600, P, 1.89 × 10^-^^5^), with negligible interstudy heterogeneity (I², 0%). DNAJC19 showed a consistent downward trend with marginal significance (SMD, −0.451, 95% CI: −0.932 to 0.030, P, 0.066). In contrast, RPS21 exhibited substantial heterogeneity (I², 74.6%) and the pooled effect did not reach significance (SMD, −0.911, 95% CI: −2.566 to 0.745, P, 0.281) (**Supplementary Figure 4**; **Supplementary Table 15**), suggesting that its expression may be modulated by study-specific confounders.

### 3.4 Predictive model based on five biomarkers demonstrates good discriminative ability

To further validate the clinical applicability of the five biomarkers, we constructed a nomogram to predict the probability of HA in the GSE46480 dataset (**Figure 3B**). Calibration analysis with 1,000 bootstrap resamples demonstrated good agreement between the nomogram-predicted probabilities and the actual observed outcomes, with a mean absolute error of 0.023, indicating that the model is well-calibrated (**Figure 3B**). The ROC curve analysis yielded an AUC of 0.95 (95% CI: 0.92-0.98), demonstrating outstanding discriminative power of the nomogram in distinguishing HA cases from healthy controls (**Figure 3C**). Furthermore, DCA revealed that the nomogram provides a positive net clinical benefit across a wide range of threshold probabilities (**Figure 3D**). These results collectively indicate that the five biomarkers, when combined into a predictive model, not only possess strong biological relevance but also exhibit robust clinical predictive performance, substantiating their potential value as diagnostic indicators for HA.

**Figure 3:**
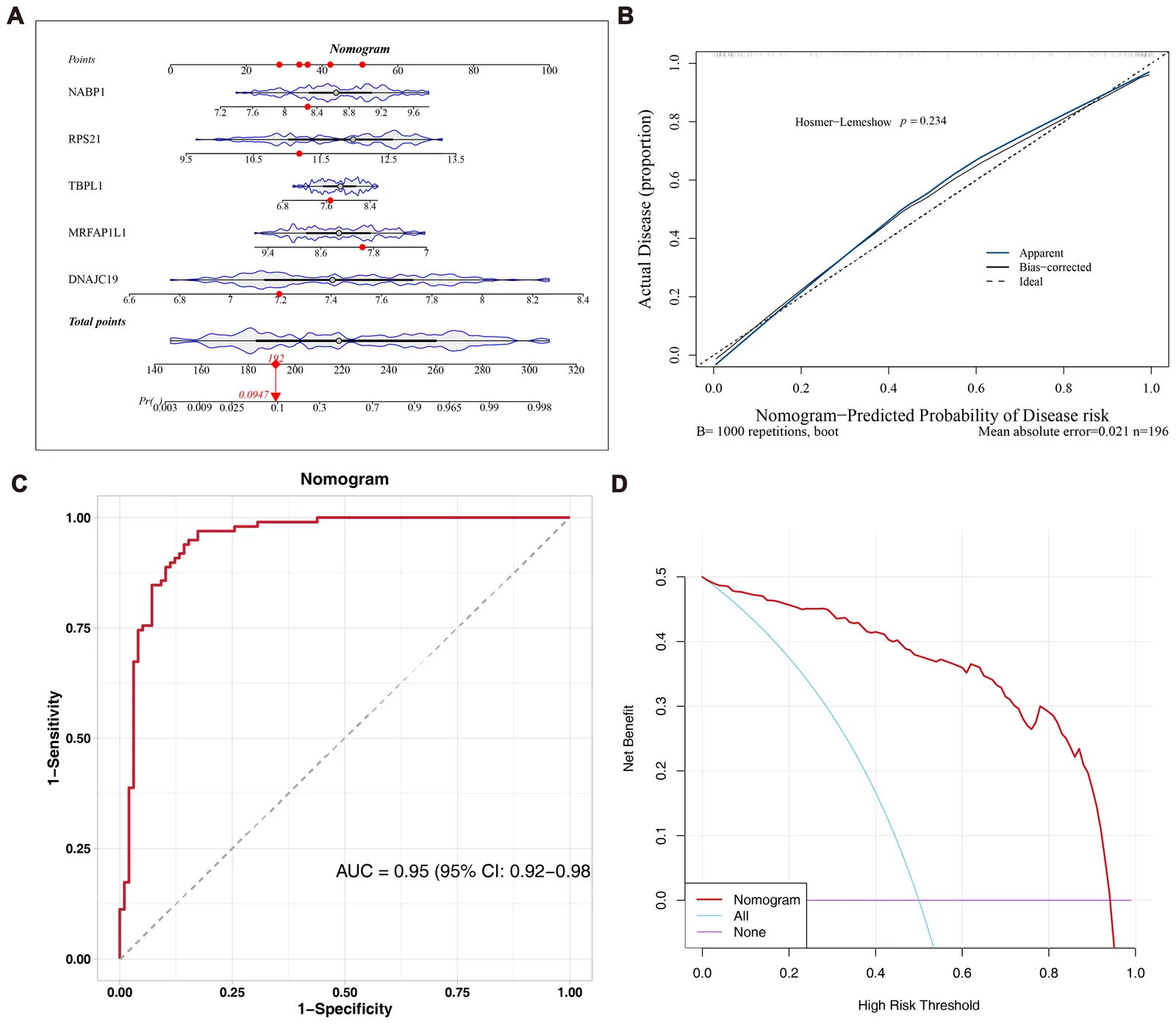
A nomogram constructed based on five biomarkers and its evaluation. **(A)** Nomogram. **(B)** Calibration curve of the nomogram. **(C)** ROC curve of the nomogram. **(D)** DCA curve of the nomogram.

### 3.5 Pathway enrichment, chromosomal localization, and GeneMANIA network analysis of candidate genes

First, in the GSEA, potential signaling pathways related to candidate genes were explored with the criteria of |NES| > 1, *p* < 0.05, and *p*.adjust < 0.05. The results showed that DNAJC19, MRFAP1L1, TBPL1, RPS21, and NABP1 were enriched in 37, 46, 45, 41, and 43 pathways, respectively (**Supplementary Tables 1**–**20**). Among these, the top five pathways enriched by all five candidate genes included ribosome, spliceosome, oxidative phosphorylation, and protein export (**Figure 4**). There were 32 core pathways commonly enriched by the five candidate genes, and the five candidate genes exhibited highly consistent trends in the magnitude and ranking of normalized enrichment scores (NES) across these 32 core pathways (**Figures 5A, B**). More importantly, significant differences in these 32 commonly enriched pathways were observed between the HA group and the control group (pathways such as apoptosis were upregulated in the HA group, and pathways such as axon guidance were downregulated in the HA group; *p* < 0.05) (**Figure 5C**). To move beyond a simple list of enriched terms, we functionally stratified these 32 shared pathways into five integrated modules based on their biological annotations: (i) energy metabolism and mitochondrial homeostasis (oxidative phosphorylation, Parkinson’s disease, Alzheimer’s disease, Huntington’s disease, dilated cardiomyopathy, arrhythmogenic right ventricular cardiomyopathy, hypertrophic cardiomyopathy, maturity onset diabetes of the young, peroxisome); (ii) protein synthesis, processing, and quality control (ribosome, protein export, proteasome, ubiquitin-mediated proteolysis, N-glycan biosynthesis); (iii) transcriptional and posttranscriptional regulation (basal transcription factors, spliceosome, and RNA degradation); (iv) genome stability and cell-cycle control (DNA replication, mismatch repair, nucleotide excision repair, cell cycle); and (v) signal transduction, membrane trafficking, and stress response (SNARE interactions, calcium signaling pathway, neuroactive ligand–receptor interaction, axon guidance, focal adhesion, ECM–receptor interaction, olfactory transduction, complement and coagulation cascades, and apoptosis). Notably, while all five biomarkers converged on these pathways, their established molecular functions suggest distinct module affinities: DNAJC19 predominated in energy-metabolic pathways, RPS21 in ribosomal and translational pathways, TBPL1 in transcriptional and splicing pathways, MRFAP1L1 in DNA-repair and cell-cycle pathways, and NABP1 in RNA-processing and nucleotide-metabolism pathways. This modular convergence implies that the five biomarkers do not merely co-occur in the same pathways, but rather occupy complementary functional niches within an integrated hypoxia-response network.

**Figure 4:**
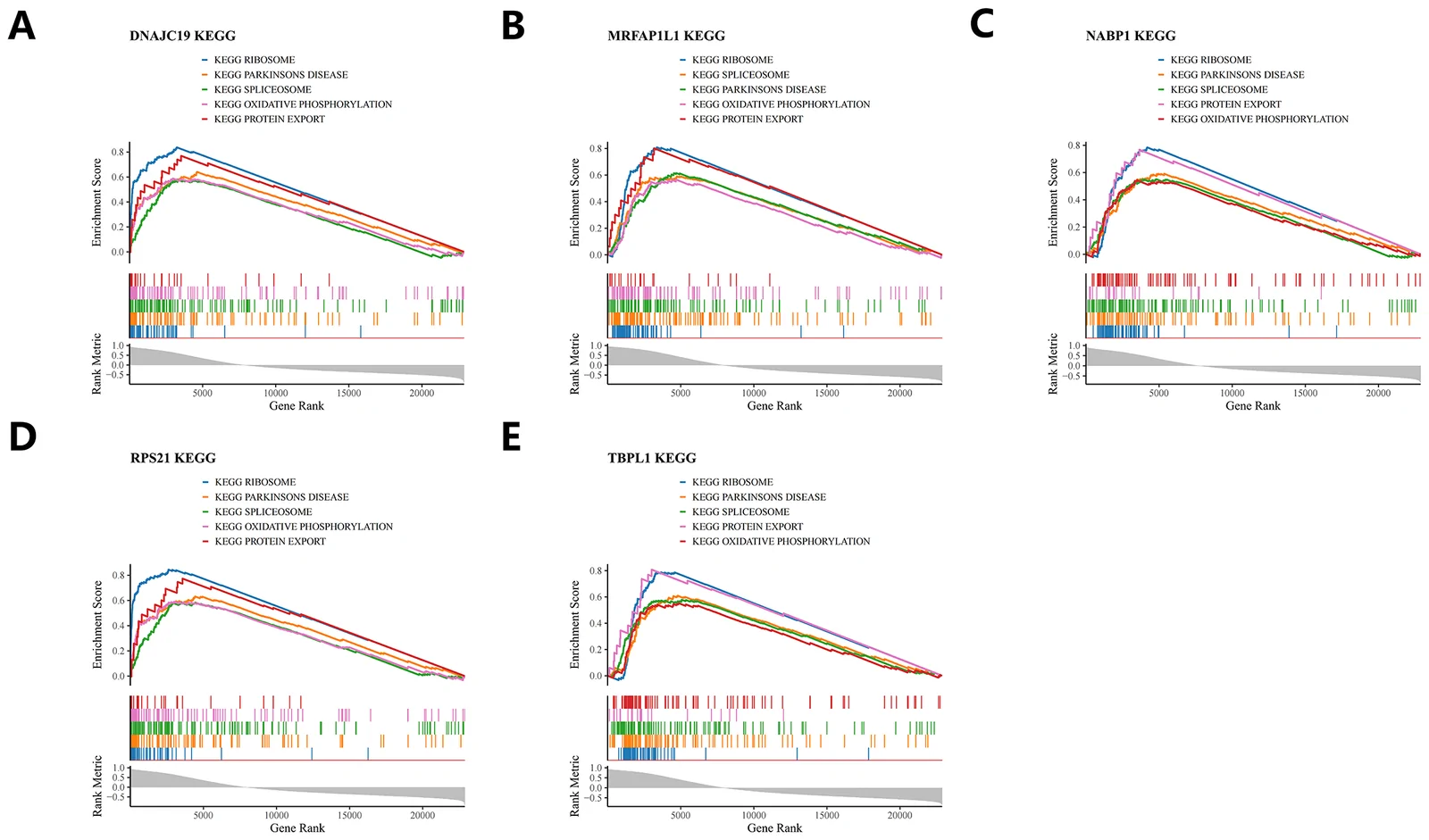
GSEA top five enriched pathways for each candidate gene. **(A)** DNAJC19; **(B)** MRFAP1L1; **(C)** NABP1; **(D)** RPS21; **(E)** TBPL1. GSEA criteria: |NES| > 1, *p* < 0.05, *p*.adjust < 0.05. Top panel: enrichment score curve of target pathway; middle panel: position of core enriched genes; bottom panel: rank metric score distribution of all genes. Peak at top-left, positive correlation with HA phenotype; peak at bottom-right, negative correlation with HA phenotype. GSEA, gene set enrichment analysis; NES, normalized enrichment score.

**Figure 5:**
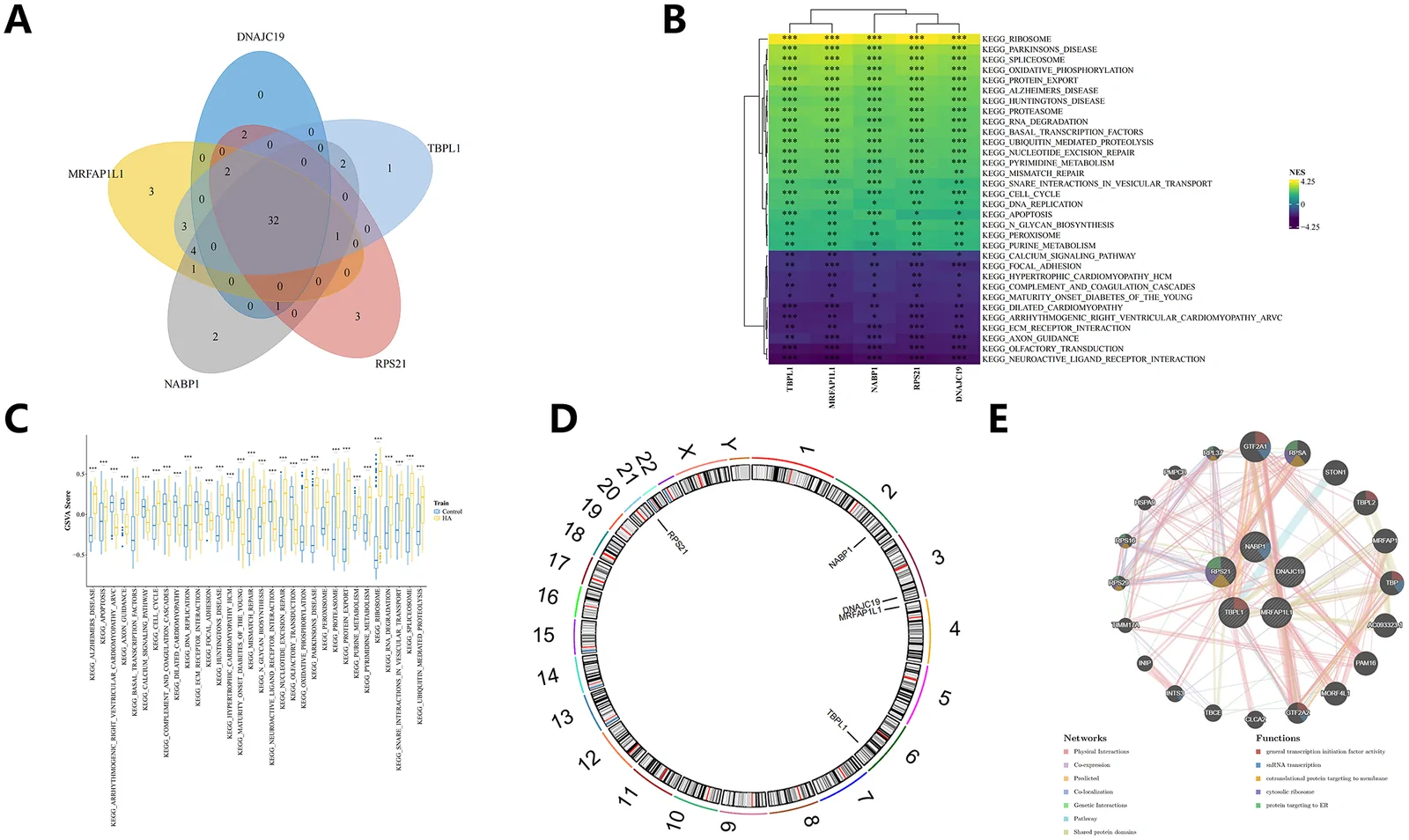
Integrated analysis of candidate genes function and localization. **(A)** Venn diagram of common enriched pathways among five candidate genes. **(B)** Cluster heatmap of normalized enrichment scores (NES) for common pathways. Color scale: red, high enrichment, blue, low enrichment. **(C)** Differential GSVA scores of common pathways between HA and control groups. **Orange, HA group, blue, control group; *p < 0.05, **p < 0.01, *p < 0.001 (Wilcoxon rank-sum test). **(D)** Chromosomal localization of candidate genes. Outer ring: human chromosome ideogram; inner lines: position of each candidate gene; gene labels are marked at corresponding loci. **(E)** GeneMANIA interaction network of candidate genes. Nodes: candidate genes and interacting genes; edge color: interaction type (physical interaction, co-expression, predicted); node color: enriched biological function. GSVA, gene set variation analysis.

Second, the chromosomal localization of candidate genes showed that NABP1, DNAJC19, MRFAP1L1, TBPL1, and RPS21 were localized on chromosomes 2, 3, 4, 6, and 20, respectively (**Figure 5D**). Meanwhile, an interaction network between candidate genes and potentially related genes was successfully constructed (**Figure 5E**). The results showed that candidate genes formed associations with 20 other human genes through multiple modes such as “physical interactions,” “co-expression,” and “predicted,” and all of them were significantly associated with functions including general transcription initiation factor activity, small nuclear (sn) transcription, cotranslational protein targeting to membrane, cytosolic ribosome, and protein targeting to endoplasmic reticulum (ER). These findings provided important clues for in-depth analysis of the molecular interaction and network regulation mechanisms of candidate genes in organisms.

### 3.6 Immune infiltration, molecular docking, and molecular dynamics simulation of candidate genes

Subsequently, immune infiltration analysis was carried out. **Figures 6A, B** shows the relative abundances of 28 types of immune cells and 21 DICs (including Gamma delta T cell, Effector memory CD4 T cell, Activated CD4 T cell, etc., with increased infiltration levels in the HA group; and Central memory CD8 T cell, Natural killer T cell, CD56dim natural killer cell, etc., with decreased infiltration levels (*p* < 0.05)). Meanwhile, as shown in **Figure 6C**, significant correlations existed among most DICs. Among them, Effector memory CD4 T cells and Activated CD4 T cells had the strongest positive correlation (cor, 0.870), whereas Central memory CD8 T cells and Activated CD8 T cells had the strongest negative correlation (cor, −0.770) (p < 0.05). As shown in **Figure 6D**, there were 16 DICs significantly correlated with DNAJC19 (|cor| > 0.3, adjusted *p* < 0.05), among which the positive correlation with Activated CD4 T cell was the strongest (cor, 0.881), and the negative correlation with Central memory CD8 T cell was the strongest (cor, −0.817); there were 17 DICs significantly correlated with MRFAP1L1, among which the positive correlation with Activated CD4 T cell was the strongest (cor, 0.905), and the negative correlation with Central memory CD8 T cell was the strongest (cor, −0.794); there were 16 DICs significantly correlated with NABP1, among which the positive correlation with Effector memory CD4 T cell was the strongest (cor, 0.806), and the negative correlation with CD56dim natural killer cell was the strongest (cor, −0.703); there were 16 DICs significantly correlated with RPS21, among which the positive correlation with Activated CD8 T cell was the strongest (cor, 0.880), and the negative correlation with Central memory CD8 T cell was the strongest (cor, −0.827); there were 17 DICs significantly correlated with TBPL1, among which the positive correlation with Effector memory CD4 T cell was the strongest (cor, 0.885), and the negative correlation with Central memory CD8 T cell was the strongest (cor, −0.731) (*p* < 0.05) (**Supplementary Table 21**). The above results showed that these candidate genes are correlated with the infiltration levels of different immune cells, and distinct correlation patterns were observed between individual candidate genes and specific immune cell types.

**Figure 6:**
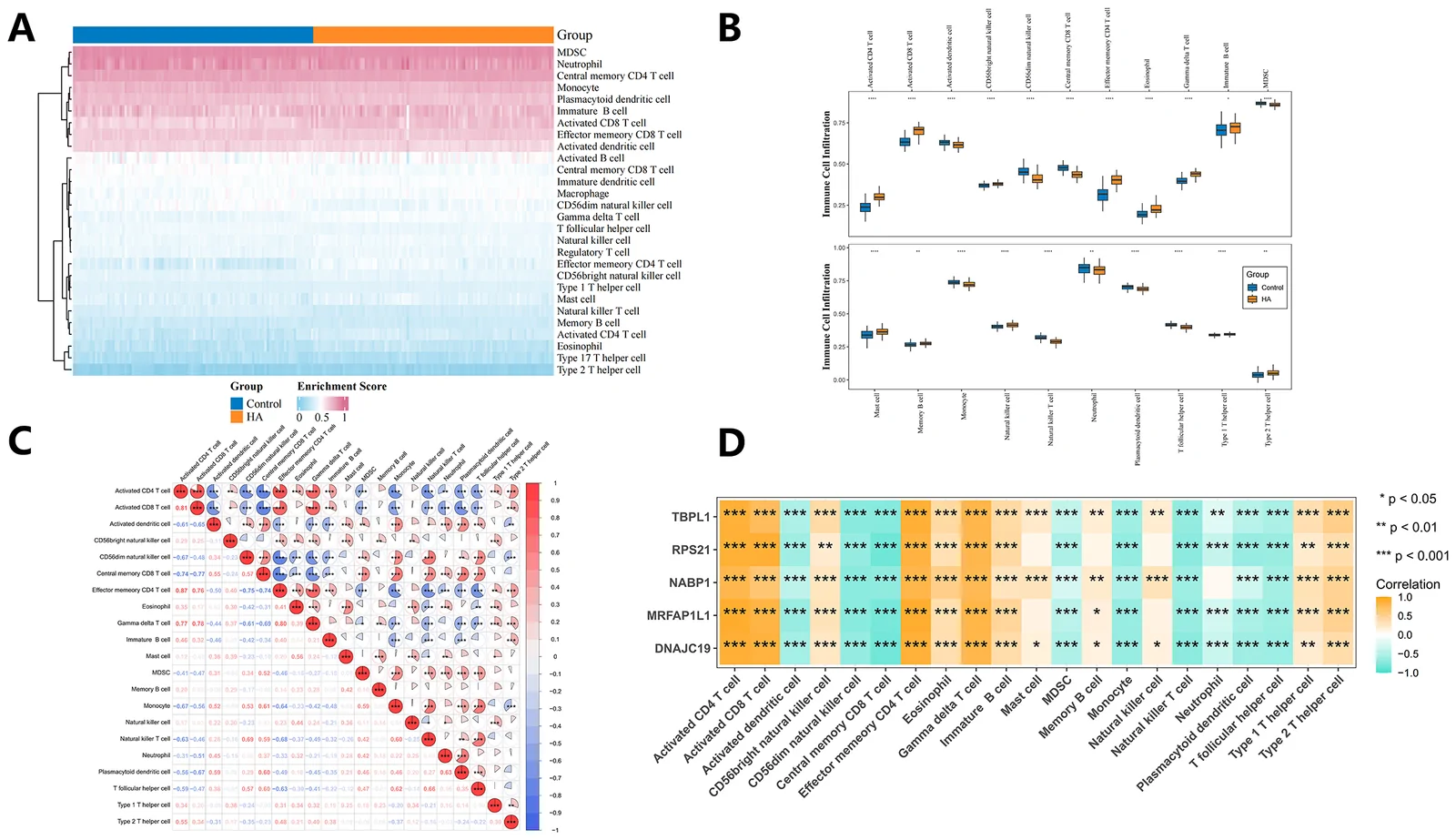
Immune infiltration analysis on the ssGSEA algorithm. **(A)** Heatmap of 28 immune cell infiltration scores. Top bar: orange, HA group, blue, control group; color scale: pink, high infiltration, blue, low infiltration. **(B)** Differential immune cell infiltration between HA and control groups. **Orange, HA group, blue, control group; *p < 0.05, **p < 0.01, *p < 0.001 (Wilcoxon rank-sum test). **(C)** Correlation heatmap of differential immune cells (DICs). **Red, positive correlation, blue, negative correlation; pie chart, correlation strength; *p < 0.05, **p < 0.01, ***p < 0.001 (BH-FDR correction). **(D)** Correlation heatmap between candidate genes and DICs. **Yellow, strong positive correlation, blue, strong negative correlation; *p < 0.05, **p < 0.01, *p < 0.001 (BH-FDR correction). ssGSEA, single-sample gene set enrichment analysis; DICs, differential immune cells; BH, Benjamini–Hochberg; FDR, false discovery rate.

Subsequently, immune infiltration analysis was performed using the ssGSEA algorithm. As shown in **Supplementary Figure 5A**, immune cells such as MDSC, Neutrophil, and Central memory CD4 T cells exhibited relatively high enrichment scores. Differential analysis identified 26 DICs between the HA and Control groups (FDR *p* < 0.05; **Supplementary Figure 5B**). The HA group showed significantly upregulated infiltration of B cells, most T-cell subsets (e.g., Activated CD4/CD8 T cells, Gamma delta T cells), NK cells, and myeloid cells, whereas Neutrophils and Th17/Th2 cells were downregulated, suggesting a Th1/Tc1-skewed immune response. Spearman correlation analysis revealed significant co-occurrence patterns among DICs (**Supplementary Figure 5C**). For instance, Activated CD4 T cells showed strong positive correlations with Activated CD8 T cells (r, 0.81) and Central memory CD8 T cells (r, 0.87), but negative correlations with Activated dendritic cells (r, −0.61). MDSCs were positively correlated with Effector memory CD8 T cells (r, 0.69) but negatively with Activated CD4 T cells (r, -0.63). Furthermore, all five key candidate genes exhibited significant correlations with DICs (FDR *p* < 0.001; **Supplementary Figure 5D**). Specifically, DNAJC19 correlated positively with adaptive immune cells (e.g., Activated B/T cells) and negatively with Neutrophils. MRFAP1L1, NABP1, RPS21, and TBPL1 showed distinct association patterns with dendritic cells, helper T cells, B cells, and myeloid cells, respectively, revealing a complex correlation network between gene expression and immune cell infiltration in HA.

Then, the molecular docking results are shown in **Table 3**. Except for RPS21 and MRFAP1L1, the binding energies of the key active components PQ-2 and polyacetylene PQ-2 with the other candidate genes (DNAJC19, NABP1, and TBPL1) were in the range of −5.2 to −6.2 kcal/mol, indicating good binding ability, which suggested that these active components may interact with the candidate genes, potentially influencing relevant biological processes. In addition, the specific docking situations are also shown in **Figures 7A–L**. The above results indicated that these active components exhibit binding potential for the corresponding candidate genes, providing preliminary molecular docking evidence for the potential interaction of relevant DFH substances in the context of HA. Finally, 100-ns molecular dynamics (MD) simulations were performed to verify the conformational stability and interaction persistence of the complexes obtained by molecular docking (**Figures 8A–D**). Consistent with the binding energy results of molecular docking, complexes with docking binding energy < −5.0 kcal/mol (DNAJC19, NABP1, and TBPL1) showed more stable structural performance in MD simulations: the RMSD values of DNAJC19-PQ-2 and DNAJC19-polyacetylene PQ-2 complexes were maintained at approximately 0.15–0.30 nm with small fluctuations, and the total system energy was stable, indicating that the binding conformation predicted by docking was reliable. The number of hydrogen bonds was closely related to the binding pocket residues: the RPS21-polyacetylene PQ-2 complex formed up to four stable hydrogen bonds at 20,000 ps, which compensated for the slightly lower docking binding energy (−4.8 kcal/mol) and maintained good binding stability. In contrast, the TBPL1-polyacetylene PQ-2 complex showed a relatively large RMSD fluctuation (0.25→1.75 nm) due to the high flexibility of the TBPL1 protein structure. MM-PBSA calculations revealed negative total binding energies for all complexes (range: −119.1 to −198.2 kcal/mol), with gas-phase energies (GGAS) as the main favorable contributor and solvation energies (GSOLV) opposing binding (**Figure 8E**). The time-series profiles showed stable total energies throughout the 100-ns simulations, with minor fluctuations, suggesting sustained complex formation. Notably, MRFAP1L1_polyacetylene_PQ-2 exhibited the strongest affinity (−198.21 kcal/mol), whereas RPS21 complexes displayed relatively weaker binding (**Figure 8**). These quantitative free-energy data corroborate the docking results and provide rigorous evidence for stable protein–ligand interactions beyond the −5.0 kcal/mol cutoff.

**Table 3:** Molecular docking results.

Candidate gene	Key active component	Binding energy(kcal/mol)
DNAJC19	PQ-2	−6.2
DNAJC19	Polyacetylene PQ-2	−5.9
MRFAP1L1	PQ-2	−5.0
MRFAP1L1	Polyacetylene PQ-2	−5.0
NABP1	PQ-2	−5.9
NABP1	Polyacetylene PQ-2	−5.4
RPS21	PQ-2	−4.8
RPS21	Polyacetylene PQ-2	−4.8
TBPL1	PQ-2	−5.8
TBPL1	Polyacetylene PQ-2	−5.2

**Figure 7:**
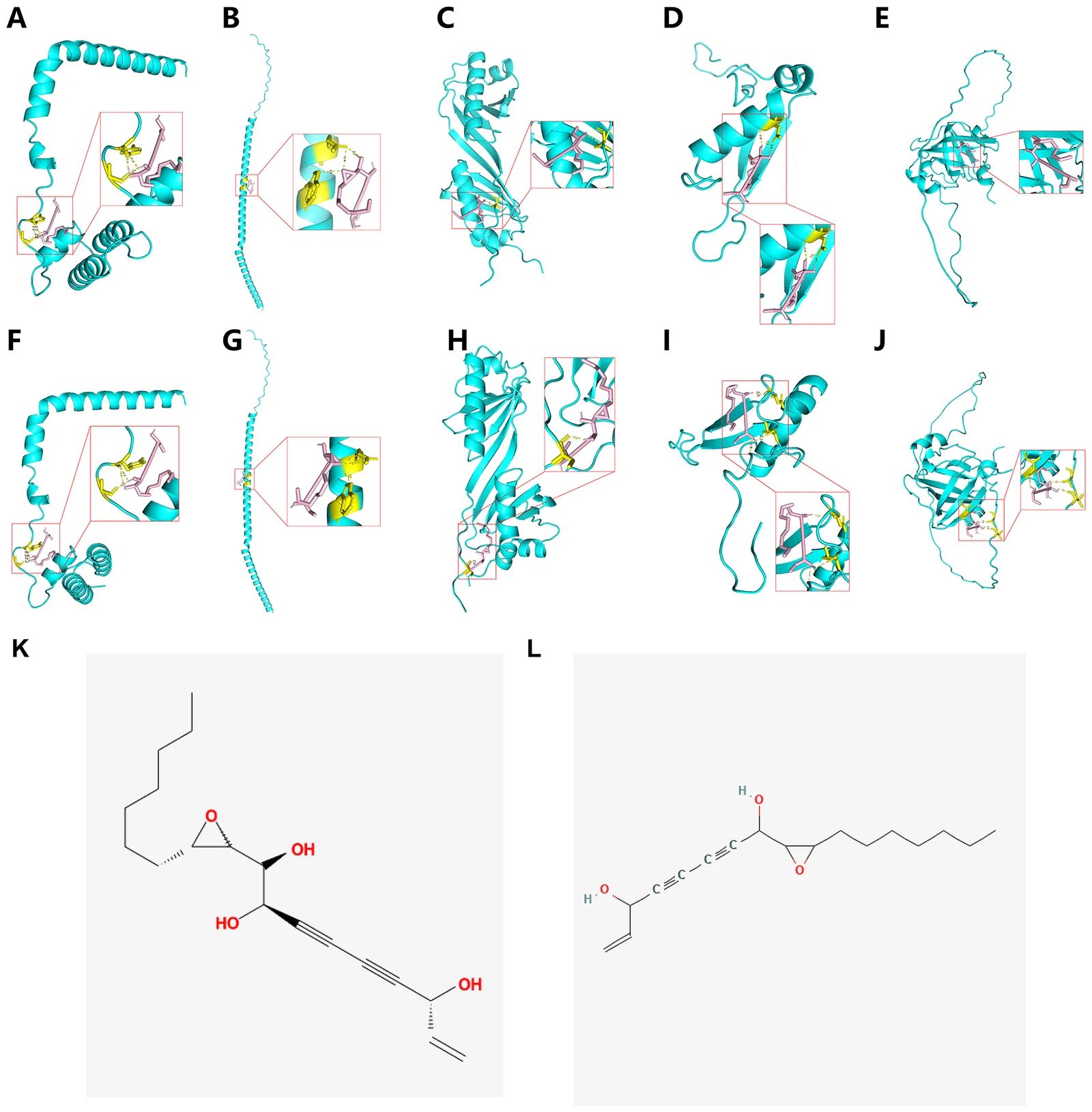
Molecular docking results of key active components with candidate genes. **(A–E)** Docking of PQ-2 with DNAJC19, MRFAP1L1, TBPL1, RPS21, and NABP1. **(F–J)** Docking of polyacetylene PQ-2 with DNAJC19, MRFAP1L1, TBPL1, RPS21, NABP1. **(K)** 2D chemical structure of PQ-2. **(L)** 2D chemical structure of polyacetylene PQ-2. Visualization: blue, protein backbone, pink, ligand (active component), yellow, key amino acid residues; yellow dashed line, hydrogen bond with length annotation. Docking tool: CB-DOCK2; binding energy < −5.0 kcal/mol indicates good binding activity.

**Figure 8:**
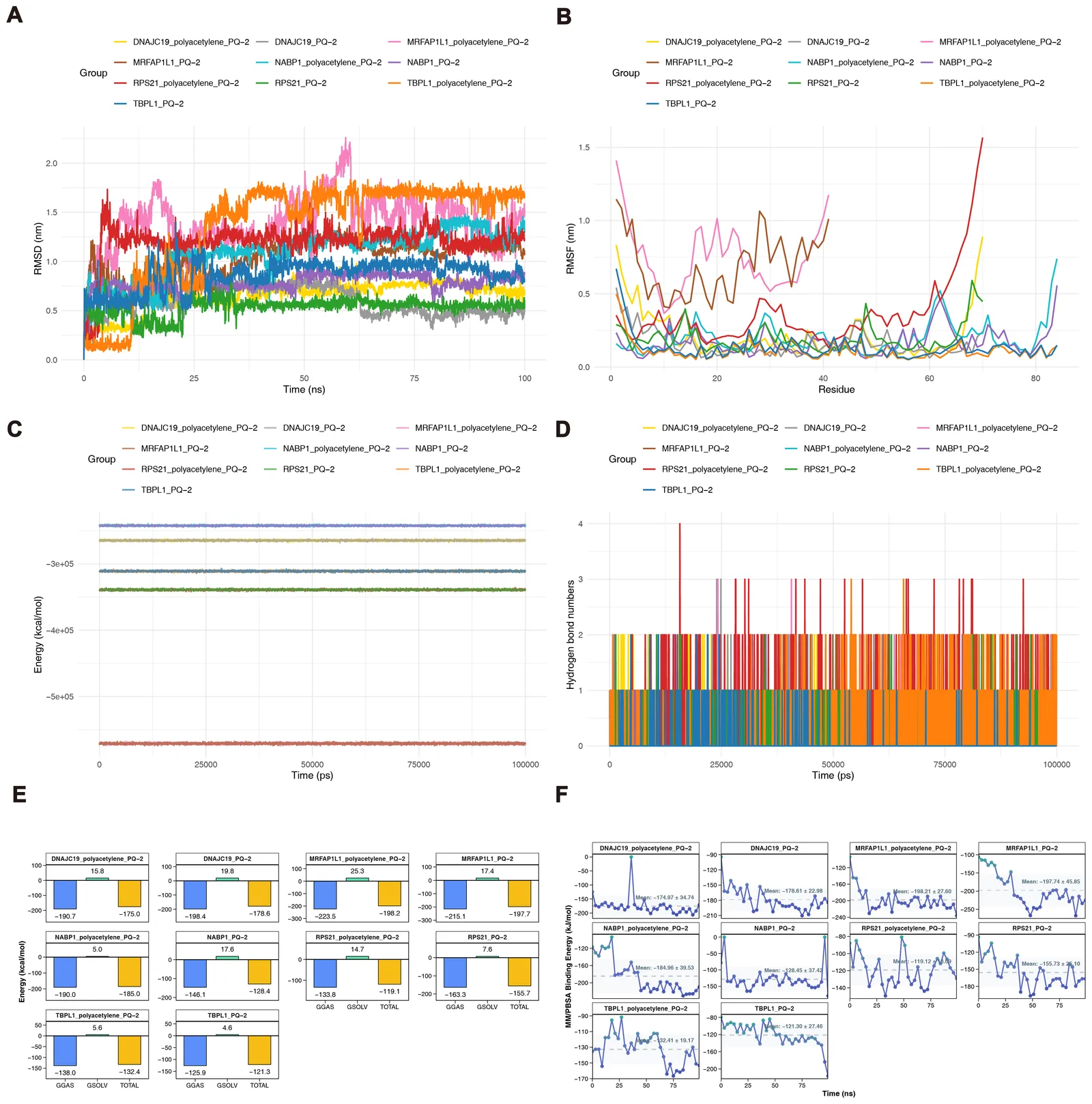
Molecular dynamics simulation analysis of the candidate gene-ligand complexes. 100-ns MD simulation was performed with AMBER14SB (protein) and GAFF (ligand) force fields; simulation system: 10 candidate gene-ligand complexes; evaluation indexes: RMSD, RMSF, total energy, hydrogen bond number. **(A)** Root mean square deviation (RMSD). X-axis: simulation time (ns), Y-axis: RMSD (nm); low and stable RMSD, high structural stability of complexes. **(B)** Root mean square fluctuation (RMSF). X-axis: protein residue number, Y-axis: RMSF (nm); reflects residue flexibility. **(C)** Total system energy. X-axis: simulation time (ns), Y-axis: total energy (kJ/mol); stable energy, stable system. **(D)** Number of hydrogen bonds. X-axis: simulation time (ns), Y-axis: hydrogen bond count; stable hydrogen bonds, strong intermolecular interaction. Different colored lines represent different complexes: DNAJC19_PQ-2, DNAJC19_polyacetylene_PQ-2, MRFAP1L1_PQ-2, MRFAP1L1_polyacetylene_PQ-2, NABP1_PQ-2, NABP1_polyacetylene_PQ-2, RPS21_PQ-2, RPS21_polyacetylene_PQ-2, TBPL1_PQ-2, TBPL1_polyacetylene_PQ-2. RMSD, root mean square deviation; RMSF, root mean square fluctuation. **(E)** The MM-PBSA free energy of dissociation and binding for each complex. GGAS represents the contribution to the gas-phase internal energy, GSOLV represents the contribution to the solvation free energy, and TOTAL represents the total binding free energy (kcal/mol); a larger negative value indicates stronger binding affinity. **(F)** MM-PBSA binding energy time series. The x-axis represents simulation time (ns), and the y-axis represents the MM-PBSA binding energy (kJ/mol); the data are labeled as the average binding energy ± standard deviation for each complex.

## 4 Discussion

HA illness represents acute stress responses to sudden exposure to HA environments. From an environmental physiology perspective, this study addresses two unresolved questions: (i) which genes and mechanisms drive hypoxia-induced immune imbalance and metabolic disruption in PBMCs, and (ii) whether bioactive DFH components can be computationally prioritized as candidates targeting these genes. The core advantage of DFH substances lies in their mild regulatory effects and the preventive concept of “treating pre-disease”. By applying these substances scientifically, it is possible to alleviate uncomfortable symptoms during HA exposure and facilitate physical recovery after returning to the plains. This approach represents a health management strategy that integrates traditional Chinese and modern HA illness medicine. In this study, bioinformatics and network pharmacology approaches screened multiple active components from *Panax quinquefolius*. Five key candidate genes—DNAJC19, MRFAP1L1, TBPL1, RPS21, and NABP1—were identified and stably bound by polyacetylene PQ-2 from *Panacis Quinquefolii Radix*. These candidate genes primarily involved in ribosomal function and oxidative phosphorylation, collectively regulating physiological and pathological states.

The five key candidate genes likely exert multilevel synergistic effects during HA adaptation. Mitochondrial dysfunction is central to HA illness pathogenesis. DNAJC19, a mitochondrial inner membrane chaperone protein (Anna et al., 2023), may be hypothesized to participate in HA illness-related pathological processes by regulating mitochondrial function, given that HA illness pathogenesis involves mitochondrial dysfunction. Additionally, RPS21, which encodes the ribosomal small subunit protein S21, is significantly downregulated upon HA exposure. This downregulation may inhibit ribosomal function, thereby impairing protein synthesis, energy metabolism, and hypoxic adaptation capacity, and correlates with early alterations in HA physiological parameters (Andrew and James, 2015). Moreover, ZCH13-mediated N6 adenosine methylation (m6A) of *NABP1* mRNA may be enhanced under hypoxic conditions, thereby influencing its stability. Indeed, the ZC3H13–NABP1 axis may mediate HA hypoxia-induced renal hypoperfusion and acute kidney injury (Qinghao et al., 2025; Si-Yang et al., 2022). Furthermore, MRFAP1L1, a nuclear protein involved in epigenetic regulation and genomic stability, facilitates transcriptional activation and DNA damage repair by stabilizing MRG15 and bridging chromatin-modifying complexes (Chong et al., 2023), suggesting a potential role in cellular stress responses under HA-induced hypoxia. Finally, TBPL1, a component of the transcription factor IID complex, is associated with the proliferation and differentiation of pulmonary fibroblasts (Hui et al., 2022), indicating its possible involvement in HA-related pulmonary fibrosis. These five candidate genes are located on distinct chromosomes (chromosomes 2, 3, 4, 6, and 20), with no overlapping genomic loci, further supporting their utility as multitarget regulatory molecules. GeneMANIA physical interaction and co-expression network analysis further revealed functional associations with 20 human genes, with collective enrichment in pathways such as “general transcription initiation factor activity” and “protein targeting to the ER (endoplasmic reticulum)”. These findings suggest that these candidate genes may form a molecular interaction network that is associated with HA-related gene expression and protein function, providing a systems-level foundation for subsequent mechanistic investigation.

The regulatory mechanisms of the five candidate genes fall into two categories: synergistic action through shared core pathways and divergent roles via distinct individual pathways. We identified 32 commonly enriched “core pathways” that were significantly altered between the HA and control groups, directly supporting their relevance to HA illness initiation and progression. These core pathways included ribosome function, oxidative phosphorylation, protein export, and spliceosome function, all of which align closely with the central pathology of HA-hypoxia-induced energy deficits and disrupted protein synthesis. Beyond these shared pathways, each candidate gene also participates in HA-related pathological mechanisms through unique pathways, reflecting high molecular complexity.

Furthermore, this study identified two key DFH active components (polyacetylene PQ-2, PQ-2) and one DFH substance (*Panacis Quinquefolii Radix*) corresponding to the five candidate gene. The resulting network revealed that the active components of *Panacis Quinquefolii Radix* can directly modulate these candidate gene, outlining a clear, multitarget pathway for natural product-based HA intervention. Unlike chemical drugs with single-target actions, the active components of DFH substances can simultaneously regulate multiple candidate gene, thereby coordinately modulating their enriched core pathways. For instance, PQ-2 can enhance energy supply via oxidative phosphorylation and suppress excessive protein synthesis through the ribosome pathway, thereby achieving multitarget, multipathway HA intervention that aligns with both the complexity of HA pathogenesis and the holistic regulatory advantage of DFH substances.

More importantly, the pathway regulation characteristics of the five candidate genes also inform the optimization of DFH-based strategies. For example, the use of active components from *Panacis Quinquefolii Radix* could address the energy deficit-oxidative damage paradox in HA by enhancing oxidative phosphorylation (i.e., increasing ATP production) and suppressing reactive oxygen species generation. In addition, modulating NABP1-mediated complement and coagulation pathway activity could alleviate excessive inflammatory responses in HA. This precision intervention based on candidate genes pathway characteristics significantly enhances the theoretical feasibility of applying DFH substances for HA prevention and treatment.

Dysregulated immune cell infiltration is a core characteristic of HA progression (Hong et al., 2023). In this study, the five candidate genes (DNAJC19, MRFAP1L1, TBPL1, RPS21, and NABP1) demonstrated significant correlations with multiple DICs, providing insights into the potential molecular mechanisms underlying HA-related immune imbalance. For example, the infiltration of proinflammatory/activated DICs such as gamma delta T cells was enhanced in the HA group, whereas that of memory/defensive DICs such as central memory CD8^+^ T cells was reduced (*p* < 0.05), consistent with the pathological manifestations of excessive inflammatory activation and weakened immune memory in HA (Robert T et al., 2023; Sylwia and Katarzyna, 2025). For instance, upregulated Activated CD4 T cells may exacerbate the release of inflammatory factors (Hao-Yun et al., 2023), potentially contributing to HA-related tissue inflammatory damage, whereas downregulated Central memory CD8 T cells could impair the body’s long-term immune defense capacity against hypoxic injury, hindering post-HA recovery and adaptation (Bing et al., 2024). Furthermore, significant correlations were observed among different DICs. Effector memory CD4 T cells showed the strongest positive correlation with Activated CD4 T cells (cor, 0.870), suggesting their potential synergistic enhancement of inflammatory responses. Conversely, central memory CD8 T cells exhibited the strongest negative correlation with Activated CD8 T cells (cor, −0.770), indicating a disrupted equilibrium between memory and activated CD8 T-cell functions in HA. These findings collectively suggest that HA-induced immune imbalance results from coordinated dysregulation across multiple cell populations.

Consistent with the immunoregulatory characteristics of HA, the five candidate genes identified in this study showed specific correlation patterns with DICs. Specifically, both DNAJC19 and MRFAP1L1 correlated with the levels of 16–17 DICs, showing the strongest positive correlation with activated CD4^+^ T cells (r, 0.881 and 0.905, respectively) and the strongest negative correlation with central memory CD8^+^ T cells (r, −0.817 and −0.794, respectively). This result suggests that these candidate genes are significantly associated with HA-associated inflammatory responses, specifically correlating with overactivation of CD4^+^ T cells and functional suppression of memory CD8^+^ T cells. For instance, DNAJC19 and MRFAP1L1 show strong positive correlations with activated CD4^+^ T-cell infiltration, which suggests a statistical association between these candidate genes and the excessive inflammatory activation observed in HA illnesses.

NABP1 and TBPL1 both exhibited strong positive correlations with Effector memory CD4^+^ T cells (r, 0.806 and 0.885, respectively), and NABP1 was strongly and negatively correlated with CD56^dim^ natural killer (NK) cells (r, −0.703). Effector memory CD4^+^ T cells mediate rapid inflammatory responses (Samuele, 2024), and their upregulation may exacerbate acute inflammatory injury in HA conditions. In contrast, CD56^dim^ NK cells possess crucial anti-infective and anti-neoplastic roles (Diana Lorena et al., 2025), and their downregulation likely compromises immune protection under HA conditions. Thus, NABP1 and TBPL1 are statistically associated with acute inflammatory activation and immune defense cell infiltration, representing potential correlational molecular markers linking these processes in HA.

RPS21 correlated with 16 DICs, displaying the strongest positive correlation with RPS21 correlated with the levels of 16 DICs. It displayed the strongest positive correlation with activated CD8^+^ T cells (r, 0.880) and the strongest negative correlation with central memory CD8^+^ T cells (r, −0.827). This pattern distinctly differs from those of other biomarkers. Given that activated CD8^+^ T cells primarily mediate cytotoxic effects (Yibin et al., 2025), their overactivation may promote cellular damage (e.g., vascular endothelial injury) in HA. These correlation patterns suggest that RPS21 is statistically associated with cytotoxic immune cell infiltration in HA, providing new avenues for exploring immune-mediated tissue damage in this condition.

In summary, through their significant associations with DICs, these HA-associated candidate genes exhibit distinct correlation patterns with multiple differential immune cells, forming a statistical correlation network linked to immune dysregulation in HA. This network encompasses inflammatory activation (e.g., activated CD4^+^ T cells), immune memory (e.g., central memory CD8^+^ T cells), and cytotoxic injury (e.g., activated CD8^+^ T cells), consistent with the complexity of HA-induced immune imbalance. These findings highlight novel molecular and cellular targets for future development of immune intervention strategies in this setting.

The core mechanism by which DFH substances such as *Panacis Quinquefolii Radix* intervene in HA-associated conditions is the binding and modulation of candidate genes by their active components. Molecular docking and 100-ns MD simulations were jointly used to verify this mechanism, and the two results were highly consistent: molecular docking predicted the binding affinity and sites, whereas MD simulations confirmed the conformational stability and interaction persistence of the complexes, providing comprehensive molecular evidence for the intervention effect of DFH substances. Specifically, molecular docking studies revealed that the key DFH active components PQ-2 and polyacetylene PQ-2 associate with DNAJC19, NABP1, and TBPL1 with binding energies in the range of −5.2 to −6.2 kcal/mol. However, no significant binding was observed with RPS21 and MRFAP1L1. MD simulations further explained the differences in structural stability among complexes: firstly, PQ-2 and polyacetylene PQ-2 are a pair of epimers with different stereochemical configurations, leading to different matching degrees with the binding pocket of candidate genes; secondly, the binding pocket of DNAJC19 is rich in hydrophobic and polar amino acid residues, which can form stable hydrogen bonds and hydrophobic interactions with ligands, so the complex has the lowest RMSD fluctuation and the best stability; thirdly, TBPL1 protein has high structural flexibility, resulting in large RMSD fluctuation of its complex, whereas RPS21 forms a stable hydrogen bond network with ligands to compensate for the low docking binding energy. These values suggest high binding affinity between PQ-2 and DNAJC19, NABP1, and TBPL1, which is required for the targeted modulation of candidate gene functions by DFH active components. For instance, PQ-2 may bind to DNAJC19 and influence its regulation of apoptotic pathways, thereby mitigating excessive cellular apoptosis in HA. Alternatively, interactions between PQ-2 and NABP1 could modulate the role of the latter in the complement–coagulation pathway, alleviating inflammatory damage associated with HA conditions. Notably, the absence of strong binding between PQ-2 and RPS21 or MRFAP1L1 may be attributed to specific structural features of these proteins such as binding pocket conformations that are incompatible with the molecular structure of PQ-2. This suggests that DFH substances may require the presence of other active components to target RPS21 and MRFAP1L1, or that structural modification of PQ-2 may be needed to enhance its binding capacity. These findings offer valuable insights for optimizing DFH active components.

Despite the systematic identification of candidate genes and key active components of drug–food homologous substances for high-altitude illness in this study, several limitations need to be addressed. First, this study is a purely computational and bioinformatic simulation; all screened candidate genes and predicted mechanisms are theoretical inferences without verification by *in vitro* cellular assays, *in vivo* animal experiments, or clinical studies, and these candidate genes cannot be regarded as clinically validated candidate genes. Second, the core candidate genes were initially screened from a single transcriptomic dataset (GSE46480), and although external validation was performed using GSE196728 and GSE133702, the statistical significance of these candidate genes varied across cohorts. This inconsistency is likely attributed to the high heterogeneity of high-altitude exposure studies, including differences in altitude gradients, exposure durations, and ethnic genetic backgrounds (Mallet et al., 2023). While RPS21 was significantly validated and other candidate genes showed consistent expression trends, large-scale, multicenter clinical cohorts are still required to verify the diagnostic value of these candidate genes. Third, this study is predominantly based on *in silico* predictions; network pharmacology, molecular docking, and molecular dynamics simulations are all *in vitro* computational models that cannot fully replicate the authentic drug–target interactions in the complex internal environment of living organisms. Although molecular docking and molecular dynamics simulations predicted the stable binding of PQ-2 to target candidate genes, further *in vitro* and *in vivo* experiments are essential to validate the specific regulatory effects of *Panacis Quinquefolii Radix* on these candidate genes. Fourth, the immune infiltration analysis was inferred from bulk peripheral blood mononuclear cell transcriptomic data using the ssGSEA algorithm, rather than direct experimental quantification of immune cell subsets. All observed correlations between the five candidate genes and differential immune cell infiltration only represent statistical associations and do not imply causal relationships. Future studies using flow cytometry for immune cell subset validation and functional experiments to explore candidate gene–immune cell interactions are needed to clarify the potential mechanistic links underlying these correlations. Fifth, MM-PBSA binding free energy calculation was not performed based on MD simulation trajectories to further quantify the binding affinity; this method can be used in future studies to more accurately verify and supplement the molecular docking results. Sixth, the diagnostic efficiency, sensitivity, and specificity of the candidate genes, as well as their precise regulatory mechanisms in high-altitude illness, remain to be further verified by targeted experiments.

## 5 Conclusion

This study predicted the potential mechanisms by which 106 DFH substances protect against hypoxia-induced injury, as well as associated promising candidate gene and signaling pathways, providing computational novel insights into the pathogenesis of HA-associated illnesses. Future work should build on these findings to focus on the candidate gene–DIC–PQ-2 axis to identify targeted intervention strategies such as modulating DNAJC19 to inhibit CD4+ T-cell infiltration or targeting RPS21. However, given the heterogeneity of high-altitude environments and the limitations of single-dataset analysis observed in our external validation, further experimental verification and multicenter clinical studies are necessary to validate and confirm these findings.

## Data Availability

The original contributions presented in the study are included in the article/**Supplementary Material**. Further inquiries can be directed to the corresponding authors.

## Supplementary material

Supplementary Figure 1 Venn diagram of 6 core DFH active components and 44 core genes.

Supplementary Figure 2 GO enrichment analysis of three sets of genes identified by machine learning methods. **(A)** GO enrichment analysis of 28 genes selected using the Boruta algorithm. **(B)** GO enrichment analysis of 21 genes selected using the LASSO algorithm. **(C)** GO enrichment analysis of 10 genes selected using the RF algorithm.

Supplementary Figure 3 Key gene expression validation analysis based on the GSE196728 **(A)** and GSE133702 (B) datasets. **(A)** The datasets were grouped by altitude: PU (3,800 m) and RI (5,100 m). **(B)** The datasets were grouped by altitude: Basal (800 m) and Altitude (4,111 m).

Supplementary Figure 4 Meta-analysis forest map of five biomarkers. **(A)** DNAJC19 **(B)** MRFAP1L1. **(C)** NABP1. **(D)** RPS21. **(E)** TBPL1

Supplementary Figure 5 Multiple testing correction analysis of immune cell infiltration and its correlation with candidate candidate genes. **(A)** Immune cell enrichment score heatmap. The top annotation bar indicates the sample groups (Orange: HA group; Blue: Control group). Each row represents a specific immune cell type. The color gradient from pink to blue indicates the enrichment score, reflecting the relative level of immune cell infiltration. **(B)** Differential immune cell infiltration between the HA and Control groups. The x-axis represents different immune cell types, and the y-axis represents the immune cell infiltration score. Statistical significance was determined by the Wilcoxon rank-sum test with FDR correction. **(C)** Correlation heatmap of differential immune cells. The x and y axes represent immune cell types. The color scale indicates the correlation coefficient, with red representing positive correlation and blue representing negative correlation. P values were adjusted by BH–FDR correction. **(D)** Correlation heatmap between key candidate candidate genes and differential immune cells. The x-axis represents immune cell types. The color intensity indicates the correlation coefficient. P values were adjusted by BH–FDR correction. Statistical significance is indicated as follows: * p < 0.05, ** p < 0.01, *** p < 0.001.

Supplementary Table 1 List of 106 drug-food homologous substances.

Supplementary Table 2 Full list of 1,103 DEGs.

Supplementary Table 3 GO enrichment results (220 terms).

Supplementary Table 4 KEGG enrichment results (20 pathways).

Supplementary Table 5 610 DFH active components (DL ≥ 0.18, OB ≥ 30%).

Supplementary Table 6 High-confidence CPI pairs from BindingDB.

Supplementary Table 7 High-confidence CPI pairs from BioSNAP.

Supplementary Table 8 High-confidence CPI pairs from KIBA.

Supplementary Table 9 Intersection of 754 high-confidence CPI pairs.

Supplementary Table 10 44 candidate genes corresponding to 6 core DFH active components.

Supplementary Table 11 Chemical identity confirmation of key active components PQ-2 and polyacetylene PQ-2.

Supplementary Table 12 28 feature genes (confirmed by Boruta) – second set.

Supplementary Table 13 21 feature genes (LASSO regression) – third set.

Supplementary Table 14 Result of common GO enrichment analyses.

Supplementary Table 15 Result of random-effects meta-analysis.

Supplementary Table 16 GSEA of DNAJC19.

Supplementary Table 17 GSEA of MRFAP1L1.

Supplementary Table 18 GSEA of NABP1.

Supplementary Table 19 GSEA of RPS21.

Supplementary Table 20 GSEA of TBPL1.

Supplementary Table 21 Detailed correlation results between candidate genes and DICs.
